# Structural and Mechanistic Studies of Measles Virus Illuminate Paramyxovirus Entry

**DOI:** 10.1371/journal.ppat.1002058

**Published:** 2011-06-02

**Authors:** Richard K. Plemper, Melinda A. Brindley, Ronald M. Iorio

**Affiliations:** 1 Department of Pediatrics, Emory University School of Medicine, Atlanta, Georgia, United States of America; 2 Children's Healthcare of Atlanta, Atlanta, Georgia, United States of America; 3 Department of Microbiology and Physiological Systems, University of Massachusetts Medical School, Worcester, Massachusetts, United States of America; 4 Program in Immunology and Virology, University of Massachusetts Medical School, Worcester, Massachusetts, United States of America; University of California San Diego, United States of America

## Abstract

Measles virus (MeV), a member of the paramyxovirus family of enveloped RNA viruses and one of the most infectious viral pathogens identified, accounts for major pediatric morbidity and mortality worldwide although coordinated efforts to achieve global measles control are in place. Target cell entry is mediated by two viral envelope glycoproteins, the attachment (H) and fusion (F) proteins, which form a complex that achieves merger of the envelope with target cell membranes. Despite continually expanding knowledge of the entry strategies employed by enveloped viruses, our molecular insight into the organization of functional paramyxovirus fusion complexes and the mechanisms by which the receptor binding by the attachment protein triggers the required conformational rearrangements of the fusion protein remain incomplete. Recently reported crystal structures of the MeV attachment protein in complex with its cellular receptors CD46 or SLAM and newly developed functional assays have now illuminated some of the fundamental principles that govern cell entry by this archetype member of the paramyxovirus family. Here, we review these advances in our molecular understanding of MeV entry in the context of diverse entry strategies employed by other members of the paramyxovirus family.

## Paramyxoviruses: Receptors and Virus Entry

The Paramyxoviridae are enveloped, non-segmented, negative-strand RNA viruses that include major human pathogens belonging to two subfamilies. The Pneumonvirinae subfamily includes respiratory syncytial virus (RSV) and the metapneumoviruses, while the Paramyxovirinae subfamily includes, amongst others, measles virus (MeV), mumps virus, human parainfluenza viruses (hPIV1-4), and the recently emerged, highly pathogenic henipaviruses Hendra (HeV) and Nipah (NiV). Members of both subfamilies are responsible for significant human morbidity and mortality. MeV, in particular, remains a major cause of childhood mortality worldwide despite the availability of a live-attenuated vaccine [1].

Of the different paramyxovirus genera, only the morbilliviruses, including MeV, and the henipaviruses are known to bind to proteinaceous receptors displayed on the surface of target cells for infection. Consequently, their attachment proteins lack neuraminidase activity, while all other members of the Paramyxovirinae subfamily carry haemagglutinin-neuraminidase (HN) attachment proteins with high specificity for sialic acid-containing oligosaccharides or glycolipids [2]. Specifically, all MeV haemagglutinin (H) attachment proteins analyzed thus far are capable of high-affinity interaction with signaling lymphocytic activation molecule (SLAM/CD150 w) [3], [4]. H proteins derived from the attenuated vaccine strain Edmonston and some isolates also bind to the regulator of complement activation (CD46) [5]–[7]. Clinically, systemic spread and viremia may be supported by a third MeV receptor that has been hypothesized to be present on epithelial cells [8], [9]. The henipavirus attachment (G) proteins have adapted to bind ephrinB2 and B3 as receptors [10]–[12].

**Figure 1:**
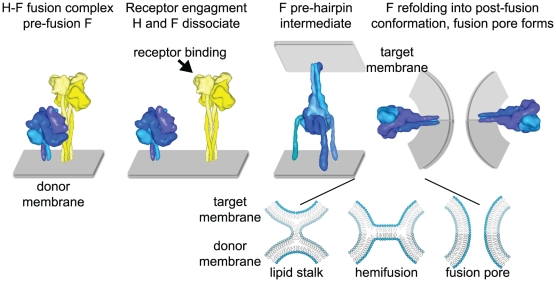
Measles virus fusion model. (Left panel) Model representation of the MeV envelope glycoprotein prefusion hetero-oligomer. The H and F complexes are aligned in a staggered head configuration in which the F head is thought to stand in contact with the H stalk [33], [57]. (Middle and right panels) Hypothetical dissociation model of F triggering. Upon binding to the cellular receptor, H and F dissociate, resulting in triggering of major conformational changes in metastable prefusion F. Refolding into the stable postfusion conformation is considered to occur through a series of intermediate conformations, including a hypothetical pre-hairpin intermediate [13], [56]. Likely, refolding of multiple F complexes is required to open a fusion pore and enable viral entry. For improved clarity, MeV H is represented as a single tetramer, and F as a single trimer in the hetero-oligomeric fusion complex. More than one F trimer may interact, however, with each individual H tetramer. The insert shows an enlarged representation of proposed lipid mixing intermediates. As F refolds, first the outer membranes are thought to fuse, creating a lipid stalk. Membrane merger is then thought to advance through hemifusion to pore formation. For clarity, F complexes have been eliminated from the lipid mixing representations. Structural renderings are based on original crystal structures (form I H head domains as in [31]), homology models of MeV F [55], [58] based on coordinates reported for pre- and post-fusion PIV5 and PIV3 F, respectively [56], [59], or hypothetical structural models (F pre-hairpin intermediate). H stalk domains are modeled in an assumed α-helical configuration [33]. High-resolution structural models were aligned at the level of the transmembrane domain (viral envelope) and then morphed into low resolution images using the Sculptor (resolution 12, voxel size 3) package [60].

All paramyxoviruses gain entry into and spread between cells by promoting direct membrane fusion. Membrane merger is mediated by the viral fusion (F) protein, which, like other class I fusion proteins such as influenza HA and HIV env, first forms metastable homo-trimers that require proteolytic activation to gain functionality [2]. Receptor binding by the attachment protein is thought to then trigger major conformational changes in mature F, resulting first in insertion of a hydrophobic domain, the fusion peptide, into the target membrane and ultimately in formation of a fusion pore through juxtapositioning of the F transmembrane domain and fusion peptide in the thermodynamically stable postfusion conformation [13]–[17] (Figure 1). Unlike retro- or orthomyxovirus entry, the complexity of the paramyxovirus fusion triggering mechanism is raised to a higher level by the fact that the receptor binding and fusion-promoting functions are contributed by separately encoded envelope glycoproteins. This physical separation of the two functions necessitates a mechanism of posttranslational linkage, which is accomplished through the formation of virus-specific hetero-oligomer complexes between the two proteins [2]. However, the overall spatial organization of functional Paramyxovirinae fusion complexes and the molecular mechanism that links receptor binding with coordinated F protein refolding into the postfusion conformation remain largely unknown.

Current evidence suggests that members of different Paramyxovirinae genera have developed distinct strategies by which the glycoprotein interaction regulates triggering of the F protein [18]–[20]. Based on endoplasmic reticulum (ER) co-retention studies with hPIV3- and PIV5-derived glycoprotein pairs, which demonstrated that an ER-retained glycoprotein mutant is unable to co-retain its unmodified counterpart [21], and the characterization of receptor binding–deficient HN proteins [22], it is thought that HN attachment proteins do not interact intracellularly with F. For paramyxoviruses that display HN, then, receptor binding and HN tetramer rearrangement appear to induce tight interaction of the HN and F oligomers at the cell surface, ultimately lowering the energy barrier for F refolding in an association model [23].

By contrast, in the case of MeV, the H-F fusion complexes appear to be pre-formed intracellularly [24]. Fusion promotion appears to follow a dissociation model, in which receptor binding results in separation of the preassembled H and F hetero-oligomers. Henipavirus G-F-mediated fusion seems to be regulated by a mechanism similar to MeV, since for both systems the level of fusion was found to be inversely correlated to the avidity of the glycoproteins for each other [25]–[27]. Also in both MeV and NiV, decreased receptor binding activity strengthens the hetero-oligomers [28], [29].

Insight into the mechanism by which the MeV H protein translates receptor binding into the activation of its homologous F protein has emerged from the recent solution of the crystal structures of H in complex with its receptors [30], [31], as well as from advances concerning the organization of MeV H-F fusion complexes [32]–[34] and predictions about H oligomer rearrangements that may take place during fusion [31]. Here, we will summarize these advances and their impact on our understanding of the mechanism of paramyxovirus fusion. In addition, we will compare the mechanism of MeV fusion triggering with that of other paramyxoviruses.

## Attachment Protein Receptors and Structure: The Structural Framework

The ectodomains of all Paramyxovirinae attachment proteins are composed of a membrane-proximal stalk, which supports a terminal globular head that mediates receptor binding. While the stalk regions are absent from all currently available crystal structures, circular dichroism analyses of PIV5 HN [35] and structure predictions for the stalks of MeV H and PIV5 HN [34], [35] support an α-helical coiled-coil configuration. It has been firmly established that the stalks of both HN and H determine F specificity [34], [36]–[38], and a domain in each that mediates the interaction with F has been identified [33], [39]. What remains unknown for any paramyxovirus attachment protein is the cascade of conformational and/or structural changes that translates receptor binding to the head region to its stalk domain, followed by triggering of F refolding.

**Figure 2:**
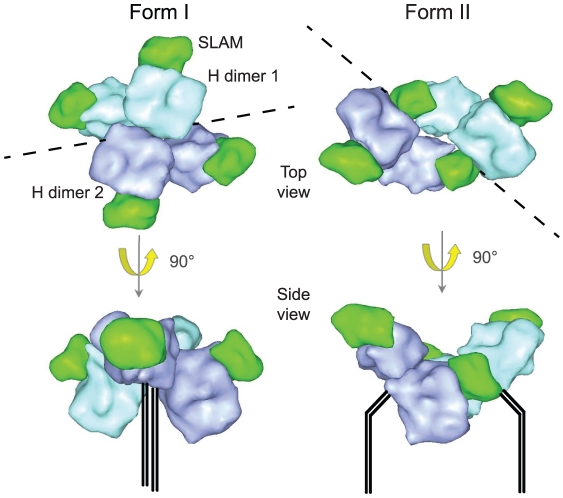
Representation of MeV H head domains complexed with soluble Slam receptor based on the coordinates reported by Hashiguchi and colleagues [**31**]. Slam moieties (dark green) and covalently linked H dimers (cyan and light purple) in the tetrameric arrangement are highlighted. Receptor binding is proposed to trigger a significant reorganization of the non-covalent dimer-dimer interface (form I versus form II [31]). In the original X-ray analysis, form II was observed when an additional L482R mutation was introduced into MeV H. This mutation was found to enhance SLAM-dependent fusion and also appeared in a clinical MeV isolate of the D1 genotype [31]. Structural renderings were prepared as described for Figure 1. Dotted lines highlight the dimer–dimer intersection. Hypothetical positions of the H stalk domains are marked in the side view representations.

Crystal structures of soluble head domains have been solved for several paramyxovirus attachment proteins, including MeV H, and reveal a common six-blade propeller fold typical of sialidase structures [40]–[44]. The HN attachment proteins interact with sialic acid through specific sites at the center of the β-propeller fold [43]–[46]. Although the H and henipavirus G proteins do not bind to sialic acid, they do both retain a vestigial central pocket analogous to the sialic acid binding pocket in HN [31], [40]–[42]. However, the two proteins have clearly adapted in different ways to be able to bind their respective receptors. While the ephrinB2/B3 binding sites in G localize to the top of the propeller and overlap with the sialic acid binding site in HN [40], [47], both known MeV receptor binding sites map to a position closer to the lateral surface of the β-propeller [30], [31] (Figure 2). Indeed, it appears that the MeV H receptor binding site must be located proximal to this position and away from the dimer interface in order to trigger fusion [48]. This use of a lateral surface of the β-propeller for receptor interaction was also confirmed by a mutational analysis of canine distemper virus H [49], which was guided by the data obtained for MeV H. Since canine distemper virus and MeV are closely related members of the morbillivirus genus, these observations suggest that lateral positioning of the receptor binding site is likely common to all morbillivirus H proteins.

In addition, H head crystals assumed an overall more cube-like structure when compared with the largely spherical folds of head domains of the related Paramyxovirina*e* HN and G proteins [41], [42]. As noted by Bowden and colleagues [40], this is consistent with: 1) the morbilliviruses and henipaviruses having adapted independently to proteinaceous receptors; and, 2) morbillivirus H being more distantly related to both HN and henipavirus G than HN and G are to each other. Experimental results indicate that a tetramer (dimer-of-dimers) may constitute the physiological oligomer for henipavirus G and several paramyxovirus HN attachment proteins [35], [44], [50]. The initial crystal structures of soluble MeV H head domains showed a monomeric or, when one of two intermolecular disulfide bonds in the H stalk domain was present, dimeric organization of the head domains [41], [42]. A more recent crystal structure of MeV H head domains complexed with CD46 confirmed the lateral position of the receptor binding site [30]. The co-crystals spontaneously assumed a homo-dimer configuration, despite the absence of stabilizing intermolecular disulfide bonds from the H head construct. This suggests that the presence of the ligand exerts a stabilizing effect on the H head arrangement. However, full-length H complexes are found in a predominantly tetrameric organization when subjected to mild-detergent extraction and native PAGE analysis [32], indicating that native MeV H, like HN, is tetrameric.

Compared to HN dimers, the MeV H head domains are twisted relative to each other in dimeric configuration and the buried protein–protein interface amounts to only approximately 1300 Å^2^, considerably smaller than the 1800–2000 Å^2^ calculated for HN. This may explain the largely monomeric nature of soluble H head domains when expressed in the absence of a stabilizing intermolecular disulfide bond. Most importantly, with respect to the mechanism of fusion, the structures of free and CD46-bound H head domains are virtually identical, arguing against receptor-induced conformational changes of the head domain as the basis for F triggering. Rather, similar to propositions for HN [44], a general spatial reorganization of the H oligomers upon receptor binding was suggested as a possible mechanism of fusion initiation. If correct, this may indeed constitute a fundamentally conserved theme of paramyxovirus entry.

The recently reported co-crystals of soluble H head domains with SLAM receptor provide groundbreaking new insight into the possible mechanism of F triggering. Unlike in previous H structures, H:SLAM co-crystals spontaneously assumed tetrameric configurations [31]. Two discrete spatial organizations were found: the first form places the four SLAM binding sites easily accessible on a relatively planar field, suggesting that all binding sites are arranged perpendicular to the viral envelope; in contrast, the second form, which was assumed by an H variant harboring an L482R mutation, shifts two of the SLAM binding sites closely into the structure (Figure 2). Form I is thought to correspond to prefusion H immediately after receptor binding, whereas form II may represent receptor-bound postfusion H that no longer interacts with F [31]. Transition between the two configurations leaves the H dimer structure itself largely intact, but results in the reorganization of the dimers relative to each other. It also involves expansion of the dimer–dimer interface (from 1312 Å^2^ in form I to 2099 Å^2^ in form II). This, in turn, would reorganize the membrane-proximal stalks from a predicted tightly grouped four-helix arrangement to an open configuration in which the stalk domains of the two H dimers are separated from one another. This dissociation of the tetrameric stalk into the two dimers then presumably releases the F protein, resulting in the triggering of the conformational changes in F by an as yet undetermined mechanism.

In another recent study, the possibility of a requirement for an alteration in the association between the monomers in each dimer in the head of MeV H was explored by the introduction of disulfide bonds across the dimer interface [48]. Such disulfide bonds eliminated the ability of the protein to trigger the homologous F protein. However, overall expression levels of this mutated H were low compared to the standard protein, complicating conclusions at this point. It has been hypothesized [51] that the disulfide bonds could prevent minor adjustments in dimer organization that may precede the significant tetramer rearrangement proposed by Hashiguchi and colleagues [31]. Interestingly, opposite results were obtained when a similar dimer stabilization approach was applied previously to the Newcastle disease virus HN protein: fusion was slightly enhanced [52].

## The Physiological MeV Fusion Complex: Mechanism of F Triggering

While the X-ray structures of partial paramyxovirus ectodomains, especially in complex with their receptors, constitute a framework for our understanding of viral entry, they lack proof that the physiological organization of native fusion complexes is accurately represented. Furthermore, little light is shed on the spatial arrangement of the functional hetero-oligomer consisting of attachment and fusion protein spikes. Interfacing structural with functional information will be required to dissect the mechanistic principles of the functional paramyxovirus fusion complex.

As discussed above, data from attachment protein chimeras and co-immunoprecipitation studies with site-directed mutants of HN and H indicate that the attachment protein stalk domains mediate the interaction with F. More recently, biochemical assessments and *in silico* alignments of H and F structures [33], [34] have suggested that the MeV attachment and fusion protein head domains are positioned at different levels relative to the viral envelope, resulting in a staggered head model (Figure 1) rather than the originally assumed lateral arrangement [33]. This model assumes an α-helical conformation of the H stalk domains, which is supported by secondary structure predictions [34], [35], mutagenesis results [33], and circular dichroism analysis of the related PIV5 HN [35]. Further experimental testing confirmed that H stalk elongations membrane-distal, but not proximal, to the proposed F binding domain are compatible with the formation of functional fusion complexes, consistent with the “staggered head” arrangement [33]. Membrane-proximal stalk extensions of up to 50% of its predicted normal length (∼60 Å of additional length in α-helical configuration) were well tolerated, arguing against direct functional contacts involving the MeV H and F head domains.

**Figure 3:**
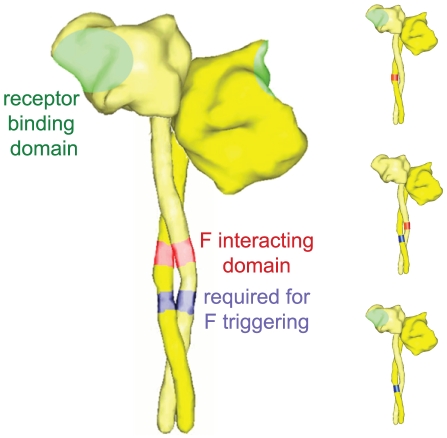
Schematic of bi-molecular H complementation to explore the organization of the physiological complex. (Left panel) Overview of previously identified functional domains in H, responsible for interaction with F [33], [34], receptor binding [29], [61], or required for F triggering [53]. For simplicity, an H dimer is shown representing form I as described in [31]. (Right panel) Co-expression of H variants defective in individual functions in all possible combinations restores F fusion promotion activity through trans-complementation of functionality [32]. Structural renderings were generated as outlined for Figure 1.

Systematic mutagenesis of a domain in the H stalk membrane-proximal to the postulated F contact zone revealed additional residues that, when mutated, block F triggering without affecting physical interaction of H and F and receptor binding [53], suggesting that receptor binding and F triggering can be separated. This was tested in a novel bi-molecular complementation assay [32] of discrete H functional defects (Figure 3), which led to three mechanistic conclusions: I) F interaction, receptor binding and F triggering constitute discrete functions that can be complemented in trans; II) efficient fusion promotion does not mandate simultaneous high-affinity docking of receptor moieties to all binding sites in an H oligomer; III) the functional H fusion oligomer is a tetramer.

Remarkably, the F-interactive domains in MeV H and NiV/HeV G may not fully overlap, since point mutations in the corresponding stalk positions of HeV G, unlike similar mutations in HN and H, do not abolish the physical interaction with F [54]. This suggests that the mechanisms used by G and H to regulate fusion may not be completely equivalent. The henipaviruses may have developed a more elaborate strategy to hold their F proteins in the metastable pre-fusion conformation in contrast to morbillivirus fusion complexes. While unknown at present, this could possibly also involve G head contacts with F in addition to the G stalk interactions.

Considering, however, that residues in the stalk domains of H, HN, and G proteins have been implicated in determining F triggering and that the disulfide backbone and hence the overall architecture of prefusion F [55], [56] are highly conserved among the Paramyxovirinae, we propose an overall largely conserved spatial organization that positions the functional paramyxovirus hetero-oligomer in a staggered head arrangement. The stoichiometry of the physiological hetero-oligomer remains unclear at present. Space constraints very likely prevent the formation of F_3_/(H_4_)_3_ or (F_3_)_4_/H_4_ complexes. However, an (F_3_)_2_/H_4_ hetero-oligomer configuration appears as a structurally plausible alternative to a simple F_3_/H_4_ arrangement. Morbillivirus- and henipavirus-derived F proteins may feature a lower inherent activation energy barrier for refolding than F proteins of parainfluenza viruses, rendering them dependent on an interaction with their attachment protein oligomer to stabilize the prefusion conformation.

Independent of an association or dissociation mechanism of F triggering, however, reorganization of the non-covalent head domain dimer–dimer interface in a tetrameric attachment protein complex upon receptor binding emerges as the common denominator among the Paramyxovirinae to transmit receptor binding to the F contact zone in the attachment protein stalk domain. Short-range changes in the microenvironment between H and F, either through receptor-induced transient association and dissociation, or receptor-induced dissociation of preassembled hetero-oligomer complexes, may then drive irreversible conformational changes in F that ultimately must result in dissolution of the hetero-oligomer and, in turn, membrane fusion.

## Summary and Perspectives

A combination of structural and functional assays has illuminated central mechanistic principles of paramyxovirus entry. Differences exist among the Paramyxovirinae with regard to morphology and relative orientation of the attachment protein head domains, position of the receptor binding site on the head β-propeller, and the strategies employed to control refolding of the mature fusion protein. However, the overall spatial organization of the paramyxovirus fusion hetero-oligomer and the transmission of receptor binding from the attachment to the fusion protein emerge as largely conserved. Receptor binding does not alter the conformation of individual H monomers but likely results in realignment of the non-covalent head domain dimer–dimer interface. By altering the attachment protein stalk configuration, the latter may change the microenvironment of the F contact zone.

Conceptually, “trigger microdomains” at the interface of functional fusion complexes constitute attractive targets for the design of novel antivirals. However, the stoichiometry of the functional hetero-oligomer, the detailed structure of the overall complex, and the molecular nature of the F residues mediating H specificity remain largely unknown, precluding structure-based drug target identification efforts. Novel approaches such as cryo-electron tomographic analysis of intact, native complexes overlayed with the available partial X-ray data in pseudoatomic structures may likely be required to address these questions. Combined with further refined functional and biochemical analyses, such procedures have the potential to advance our molecular insight into the organization and functional foundation of the fusion complex to a degree where *in silico* identification of druggable sites for the development of future therapeutics and prophylactics becomes meaningful.
